# Using machine learning algorithms to predict COVID-19 vaccine uptake: A year after the introduction of COVID-19 vaccines in Ghana

**DOI:** 10.1016/j.jvacx.2024.100466

**Published:** 2024-02-27

**Authors:** Cornelius C. Dodoo, Ebo Hanson-Yamoah, David Adedia, Irene Erzuah, Peter Yamoah, Fareeda Brobbey, Constance Cobbold, Josephine Mensah

**Affiliations:** a School of Pharmacy, University of Health and Allied Sciences, Ho, Ghana; b School of Basic and Biomedical Sciences, University of Health and Allied Sciences, Ho, Ghana; c University of Ghana Medical Centre, Accra, Ghana; d Cape Coast Teaching Hospital, Cape Coast, Ghana

**Keywords:** Vaccine hesitancy, COVID-19 vaccine, Machine learning

## Abstract

The impact of vaccine hesitancy on global health is one that carries dire consequences. This was evident during the outbreak of the COVID-19 pandemic, where numerous theories and rumours emerged. To facilitate targeted actions aimed at increasing vaccine acceptance, it is essential to identify and understand the barriers that hinder vaccine uptake, particularly regarding the COVID-19 vaccine in Ghana, one year after its introduction in the country.

We conducted a cross-sectional study utilizing self-administered questionnaires to determine factors, including barriers, that predict COVID-19 vaccine uptake among clients visiting a tertiary and quaternary hospital using some machine learning algorithms. Among the findings, machine learning models were developed and compared, with the best model employed to predict and guide interventions tailored to specific populations and contexts. A random forest model was utilized for prediction, revealing that the type of facility respondents visited and the presence of underlying medical conditions were significant factors in determining an individual's likelihood of receiving the COVID-19 vaccine. The results showed that machine learning algorithms can be of great use in determining COVID-19 vaccine uptake.

## 1 Introduction

Vaccines and vaccination remain the most cost-effective components in primary healthcare settings[18]. Since the successful implementation of the world’s first vaccine in 1796, vaccines have played important roles in preventing the spread of communicable diseases throughout the world and in some instances, leading to their complete eradication[16]. In an assessment by Rappuoli et al. [32], it was theorized that vaccines prevent a minimum of 2.5 million deaths per year approximately saving about five lives every other minute. Despite vaccines being considered an innovative strategy in safeguarding the health of populations across the globe, vaccine-preventable diseases continue to be a leading cause of morbidity and mortality especially in children under the age of 5 years[17].

Before the emergence of Coronavirus disease 2019 (COVID-19), vaccine preventable deaths were largely attributed to *Streptococcus pneumoniae*, Rotavirus, *Bordetella pertussis*, Measles virus, *Haemophilus influenzae* type b (Hib) and Influenza virus [17]. Even with the widespread coverage of vaccines, limited epidemics of vaccine-preventable diseases such as mumps and polio have been reported in several countries around the world [31], [35].

The successes of vaccines and vaccination campaigns in eradicating and preventing infectious diseases have not been without controversy. Misinformation and conspiracy theories, limited access to vaccines, religious and philosophical beliefs, fear and mistrust of the medical establishment have been conceived to be the barriers to vaccine uptake throughout the years[24]. Concerns about possible connections between vaccines and autism have been widely reported by various populations. Although scientific papers and evidence have repeatedly proven that cases of autism and vaccines are unrelated, doubts persist[14].

Despite the overwhelming advantages of vaccination, vaccine uptake continues to decline worldwide as a result of inaccurate information on vaccines by conspiracy theorists, a lack of confidence in the safety of vaccines, inaccessibility of vaccines and vaccine hesitancy and refusal [12]. The COVID-19 pandemic has underscored the essential role that vaccination and vaccine uptake play in limiting the spread of infectious illnesses. However, COVID-19 vaccines like many other vaccines, were associated with hesitancy for several reasons including those mentioned earlier for other vaccines. COVID-19 vaccine hesitancy varied widely between countries and between groups with different sociodemographic characteristics, however it appeared to be higher in low- and middle-income countries than developed countries [23], [34]. In a study by Brackstone et al. [8], COVID-19 vaccine hesitancy was 52.2 % in June 2022 and this was attributed to not having enough vaccine-related information and concerns over vaccine safety. In another similar study in Ghana, the predictors of unwillingness to participate in COVID-19 trials and uptake of COVID-19 vaccines were married persons, females, Muslims, older persons, residents of less urbanized regions and persons with lower or no formal education [3]. Reports of blood clotting following the administration of some vaccines also created a cautious atmosphere among the population[2], [6].

In order to guide targeted interventions that can increase vaccine acceptance, it is necessary to recognize and comprehend the barriers that prevent vaccine uptake. This study sought to determine the barriers to COVID-19 vaccine uptake amongst clients visiting a teaching and quaternary hospital in Ghana and develop a model that can predict and guide interventions tailored to specific populations and contexts using some machine learning algorithms. Machine learning algorithms have been employed in several studies that focused on COVID-19. Oyewola et al. [29] used some machine learning algorithms to determine COVID-19 vaccine acceptance in countries where residents were vaccinated. Also, Osman and Sabit [28] used some machine learning algorithms in determining vaccination rates among states in the United States of America.

## 2 Method

### 2.1 Study design and study site

A cross-sectional study using self-administered questionnaires was used to determine some barriers to COVID-19 vaccine uptake in Ghana a year after the introduction. A convenience sampling technique was employed in the recruitment of participants. The survey was conducted from May to July 2022 at University of Ghana Medical Centre (UGMC) and Cape Coast Teaching Hospital (CCTH). UGMC is a quaternary based healthcare facility with a 1000-bed capacity. The facility has over 40 departments including General Surgery, Cardiothoracic, Obstetrics & Gynaecology, Paediatrics, Pharmacy, Internal Medicine, Emergency, Laboratory, Critical Care, Public Health and Imaging. It serves as the referral site for many government and private hospitals across Ghana. It also serves as a referral hospital for many other countries in the West African Subregion. CCTH is a 400-bed capacity referral and teaching hospital situated in the central region of Ghana. It provides out-patient and in-patient general and specialized services in diagnostics and rehabilitation. The departments of the hospital have been grouped into sub-business management centers which include Internal medicine, Maternal Health, Paediatrics, Surgery, Critical care, Accidents and Emergency, Diagnostics and Imaging, Pharmacy. The facility also serves as a training site for undergraduate and post-graduate students from various medical institutions.

### 2.2 Sample size determination

Applying the sample size computational method employed by Cochran [10] for a cross-sectional study, using a sampling error of 2.5 %, a confidence level of 95 % and a proportion of population of 95.2 %, the minimum sample was calculated as 281.

### 2.3 Study population and recruitment

Individuals seeking clinical care at the Out Patient Department of the two facilities were recruited as participants upon visiting the facility. Participants were fully briefed about the nature and scope of the study. Participants who met the inclusion criteria were made to sign consent forms before proceeding to the survey. Individuals visiting either facility above 18 years and willing to partake in the study were included. Individuals visiting either facility demanding emergency attention were excluded.

### 2.4 Statistical analysis

The results were reported in tables and figures, data were organized as frequencies and percentages. Chi-square test of association was used to assess bivariate associations between vaccine uptake and factors. A logistic regression was used to determine the factors of vaccine uptake. The Nagelkerke’s R squared was used to assess the model, however, it does not show the percent of variance explained but only the correlation between the dependent and predictors. Also, the logistic regression model cannot identify non-linear relationships and it is non-iterative as well as its dependence on assumptions. As a result, some machine learning algorithms were used to train models with 80 % of the dataset and 20 % for testing the models, since these algorithms can over fit the model with the data used. The generalized linear model, k nearest neighbors and random forest algorithms were employed in this study.

### 2.5 Ethics

Ethical clearance was obtained from the University of Health and Allied Sciences Research and Ethics Committee (UHAS- REC A.7 [22] 21 – 20, UHAS-REC A.7 [16] 21–22), the UGMC Institutional Review Board (UGMC-IRB/MRSC/0003/2022) and CCTH Ethics Review Committee (CCTHERC/EC/2022/093).

## 3 Results and discussion

### 3.1 Demography and other characteristics

Demographic data of the participants was obtained, and a correlation between these factors and the influence of COVID-19 vaccine uptake was determined (Table 1). About 66 % of the participants were recruited from CCTH whilst the remaining (34 %) were from UGMC. The majority of the participants (40.8 %) were between the ages of 18 and 29 years, 64.3 % were females, 82.3 % were Christians, 47.6 % were single and 47.3 % were married. Also, 66.2 % had tertiary education, 59.2 % were employed, and 39.2 % had health insurance.

**Table 1:** Demography and correlates of vaccination

		COVID - 19 Vaccine Uptake				
Characteristics		Yes N (%)	No N (%)	Total (%)	p-value	COR (95 %CI)
**Age**	18–29	33(30.3)	94(46.5)	127(40.8)	ref	
	30–39	41(37.6)	53(26.2)	94(30.2)	0.006	2.2(1.25,3.89)
	40–49	8(7.3)	30(14.9)	38(12.2)	0.537	0.76(0.32,1.82)
	50–59	19(17.4)	20(9.9)	39(12.5)	0.007	2.71(1.29,5.69)
	> 60	8(7.3)	5(2.5)	13(4.2)	0.007	4.56(1.39,14.92)
**Gender**	Male	36(33)	75(37.1)	111(35.7)	0.471	0.84(0.51,1.36)
	Female	73(67)	127(62.9)	200(64.3)	ref	
**Facility**	UGMC	69(63.3)	38(18.8)	107(34.4)	<0.001	7.45(4.4,12.59)
	CCTH	40(36.7)	164(81.2)	204(65.6)	ref	
**Religion**	Christian	87(79.8)	169(83.7)	256(82.3)	ref	
	Muslim	16(14.7)	30(14.9)	46(14.8)	0.916	1.04(0.54,2)
	None	6(5.5)	3(1.5)	9(2.9)	0.070	3.89(0.95,15.91)
**Marital Status**	Single	47(43.1)	101(50)	148(47.6)	0.511	0.85(0.52,1.38)
	Married	52(47.7)	95(47)	147(47.3)	ref	
	Divorced/Widowed	10(9.2)	6(3)	16(5.1)	0.034	3.05(1.05,8.85)
**Education**	Tertiary	75(68.8)	131(64.9)	206(66.2)	ref	
	SHS	29(26.6)	48(23.8)	77(24.8)	0.846	1.06(0.61,1.81)
	JHS	5(4.6)	23(11.4)	28(9)	0.052	0.38(0.14,1.04)
**Occupation**	Employed	72(66.1)	112(55.4)	184(59.2)	0.069	1.56(0.96,2.54)
	Unemployed	37(33.9)	90(44.6)	127(40.8)	ref	
**Insurance status**	Health Insured	51(46.8)	71(35.1)	122(39.2)	0.045	1.62(1.01,2.61)
	None	58(53.2)	131(64.9)	189(60.8)	ref	

Ref: reference.

Among the participants surveyed, 35 % declared that they had taken the vaccine. Out of the number, 61 % were fully vaccinated whilst 39 % were partially vaccinated at the time of data collection. Among those who had not taken the vaccine, 63.1 % were unlikely to do so, 22.8 % were undecided whilst 14.1 % were likely to take the vaccine.

Age, location, marital status and health insurance status were significantly associated with vaccine uptake. Individuals within age groups of 30–39 (p-value = 0.006), 50–59 (p-value = 0.007) and more than 60 years (p-value = 0.007) were more likely to take vaccines than those within 18–29 years. These findings are consistent with a study by[13]which concluded that participants between 18 and 29 were less likely to get vaccinated compared to those who are 65 years and older. When quizzed about the reasons for low uptake rates within their age bracket, perceived long-term or unknown risks of side effects and perceived disadvantages were identified as the main deterrents to vaccination [36].

Marital status as described by Ang et al. [5] is as an independent factor associated with vaccine uptake. Ang et al. [5] inferred that those who are married are less likely to receive vaccines. Respondents from UGMC (p-value = 0.006), individuals who were divorced or widowed (p-value = 0.034) and those with health insurance (p-value = 0.045) were more likely to take vaccines than those within CCTH, those who are married and those without health insurance, respectively. This aligns with Ang et al. [5]’s study which has showed that single status (separated/divorced/widowed) individuals are more likely to get the vaccine. However, findings from Almotairy et al. [4] and Abbas et al. [1] contradict this conclusion. These studies stated that married individuals were more likely to be vaccinated than those who were singles (separated/divorced/widowed). Overall, data from UGMC and CCTH study sites support previous research suggesting that vaccination coverage is significantly lower among adults without health insurance compared with those with health insurance [25].

A correlation of identified COVID −19 related factors was also obtained (Table 2). Participants with underlying conditions (p-value < 0.001) and history of COVID-19 infection (p-value < 0.001) were less likely to take vaccines than those who did not have any underlying conditions and those who never had COVID-19 infection (Table 2). This observation is consistent with a study conducted in the United States of America which found that individuals with past diagnosis of COVID-19 were less likely to get vaccinated[27].

**Table 2:** COVID-19 related factors and correlates on vaccination

		COVID - 19 Vaccine Uptake				
Characteristics		Yes N (%)	No N (%)	Total (%)	p-value	COR (95 %CI)
**Underlying Conditions**	Present	43(39.4)	150(74.3)	193(62.1)	<0.001	0.23(0.14,0.37)
	Absent	66(60.6)	52(25.7)	118(37.9)	ref	
**Family Member with COVID-19 Infection**	Positive	43(39.4)	45(22.3)	88(28.3)	0.001	2.27(1.37,3.78)
	Negative	66(60.6)	157(77.7)	223(71.7)	ref	
**Source Group**	1	34(31.2)	96(47.5)	130(41.8)	<0.001	0.32(0.17,0.61)
	2–3	43(39.4)	77(38.1)	120(38.6)	0.032	0.51(0.27,0.95)
	>3	32(29.4)	29(14.4)	61(19.6)	ref	
**Previous COVID-19 Infection**	Yes	57(52.3)	153(75.7)	210(67.5)	<0.001	0.35(0.21,0.58)
	No	52(47.7)	49(24.3)	101(32.5)	ref	

Ref: reference.

Family history of infection has been positively linked to as a potential reason for vaccination. A systematic review study by Kessels et al. [21] discovered that teenage girls with family history of sexually transmitted infections (STIs) or HPV-related diseases were more likely to be vaccinated against HPV. Likewise, in a publication by Resende et al. [33] on the concerns regarding hepatitis B immunization among dentists in Brazil, it was observed that individuals with a familial background/history of hepatitis B had a higher tendency to receive vaccination against the ailment. Based on data from UGMC and CCTH, it was noted that participants who had a family member infected with COVID-19 were more likely to take vaccines than those without a family member with COVID-19 infection (p-value = 0.001). This was in conformity with results from a study by Elhadi et al. [15] which concluded that probability of accepting the COVID-19 vaccine was higher among individuals who reported having a family member or friend who contracted the virus. Individuals who expressed the likelihood of taking the vaccine stated that they did so in order to protect themselves and their family members.

Individuals with greater access to health information usually have been documented to have increased vaccination uptake rates compared to those with less access as per Jung et al. [19]. Participants with one (p-value < 0.001), or two to three (p-value = 0.032) sources of information on COVID-19 vaccines were less likely to take vaccines as compared to participants with more than three sources of information on COVID-19 vaccine. Comparably, an analysis by Kulkarni et al. [22] revealed that increased access to multiple sources of information was linked to high rates of vaccine uptake thereby suggesting that acquiring information from varied sources can potentially impact vaccination behaviours and further affecting vaccine uptake rates.

Barriers such as the belief that COVID-19 vaccine may cause an infection, and peer and family pressure were associated with vaccine uptake (Table 3). Participants who agreed that the COVID-19 vaccine may cause infection (p-value < 0.001) were less likely to get vaccinated than those who disagreed. Hence this finding proved that individuals who do not hold negative perceptions about the safety of vaccines are more inclined to accept and receive it [7], [20].

**Table 3:** Association between barriers to vaccination and vaccine uptake

	COVID-19 Vaccine Uptake				
		YES	NO	p-value	COR (95 % CI)
If I decided to get the COVID-19 vaccine, it would be hard to find a provider or clinic	Agree	56	87	0.161	1.4(0.88,2.23)
	Disagree	53	115		
The COVID-19 vaccine might have side effects	Agree	95	187	0.117	0.54(0.25,1.17)
	Disagree	14	15		
The COVID-19 vaccine may cause infection	Agree	63	154	<0.001	0.43(0.26,0.7)
	Disagree	46	48		
The COVID-19 vaccine may not be effective	Agree	46	102	0.162	0.72(0.45,1.15)
	Disagree	63	100		
I am not sure whether or not I have to get the vaccine	Agree	55	103	0.929	0.98(0.61,1.56)
	Disagree	54	99		
I don't have time to get the vaccine	Agree	42	76	0.875	1.04(0.64,1.68)
	Disagree	67	126		
	Disagree	78	144		
My religious background prevents me from taking the vaccine	Agree	32	51	0.434	1.23(0.73,2.07)
	Disagree	77	151		
My health condition prevents me from vaccine	Agree	36	77	0.373	0.8(0.49,1.31)
	Disagree	73	125		
Peer and family pressure prevents me from taking the vaccine	Agree	22	66	0.020	0.52(0.3,0.91)
	Disagree	87	136		

Brewer et al. [9] noted that it is common for individuals to seek opinions from their social network, including family members, friends and acquaintances, when considering their attitudes towards vaccination. This can result in the inclusion of vaccination decisions into their social identity in order to fit in. However, respondents of this survey who agreed (p-value = 0.020) that peer and family pressure prevented them from taking the vaccine were less likely to be vaccinated than those who disagreed with it. This also was in contrast with a study by Wilson et al. [37], which theorized that interpersonal relationships could play a role in vaccination campaigns. It was revealed that individuals who were uncertain about getting vaccinated often turned to trusted friends or family members for advice. The influence of peers could either have a positive or negative impact on one's decision to get vaccinated. When peers chose not vaccinate or expressed negative attitudes toward vaccination, it often influenced individual's decision not to get vaccinated. Further, in the case of the HPV vaccine, it was noticed that vaccine uptake was higher for individuals who reported interactions from family and friends about it[21].

Barriers such as difficulty finding a provider or clinic, fears about COVID-19 vaccine causing infection and doubts about the effectiveness of COVID-19 vaccine were not associated with vaccine uptake. Yet, when the data was stratified by gender, they showed association with vaccine uptake among males. Individuals who had difficulty finding a provider or clinic were more likely to get vaccinated (COR: 2.35, CI: 1.04, 5.29). Those who agreed COVID-19 vaccine may cause infection (COR: 0.26, CI: 0.11, 0.64) and those who perceived the COVID-19 vaccine as ineffective (COR: 0.28, CI: 0.12, 0.65) were less likely to take vaccine. Furthermore, the current health conditions of participants did not show association with vaccine uptake, however, when the data were stratified by gender, it showed association with vaccine uptake among females. Females with health conditions were less likely to get vaccinated (COR: 0.52, CI: 0.27, 0.99) (Table 4). This observation is consistent with other studies that have reported lower vaccine uptake among females compared to males[11], [26], [30].

**Table 4:** Predictors of vaccine uptake

	Predictors	AOR (95 % CI)	p-value
	(Intercept)	1.08(0.24, 4.88)	0.919
Age (Ref: 18–29)			
	30–39	2.22(0.99, 5.07)	0.055
	40–49	0.49(0.14, 1.50)	0.225
	50–59	1.45(0.47, 4.37)	0.511
	> 60	2.68(0.49, 15.35)	0.255
Gender (Ref: Female)			
	Male	0.88(0.47, 1.64)	0.689
Marital Status (Ref: Married)			
	Divorced/Widow	1.51(0.36, 6.53)	0.571
	Single	1.43(0.71, 2.93)	0.320
Education (Ref: Tertiary)			
	JHS	0.52(0.14, 1.67)	0.296
	SHS	1.08(0.55, 2.12)	0.822
Occupation (Ref: Not working)			
	Working	1.06(0.57, 1.98)	0.858
Insurance (Ref: Uninsured)			
	**Insured**	**2.33(1.25, 4.43)**	**0.009**
Underlying conditions (Ref: No)			
	**Yes**	**0.38(0.18, 0.84)**	**0.015**
Unavailable provider (Ref: Disagree)			
	Agree	0.87(0.48, 1.56)	0.648
Side effects (Ref: Disagree)			
	Agree	0.49(0.17, 1.37)	0.173
Cause infection (Ref: Disagree)			
	Agree	0.72(0.36, 1.47)	0.357
Ineffective (Ref: Disagree)			
	Agree	0.6(0.32, 1.11)	0.108
Indecision (Ref: Disagree)			
	Agree	0.71(0.33, 1.50)	0.377
No time (Ref: Disagree)			
	Agree	1.31(0.60, 2.84)	0.491
Religious background (Ref: Disagree)			
	**Agree**	**4.18(1.39, 13.04)**	**0.012**
Health condition (Ref: Disagree)			
	Agree	0.46(0.20, 1.01)	0.059
Peer pressure (Ref: Disagree)			
	Agree	0.77(0.36, 1.61)	0.496
Location (Ref: CCTH)			
	**UGMC**	**3.57(1.47, 8.96)**	**0.006**

A logistic regression with R package reported a significant model (χ^2^ = 83.387, p-value < 0.001), with Cox and Snell, as well as Nagelkerke pseudo-R squared values of 0.24 and 0.32, respectively. This showed that the vaccine uptake has appreciable correlations with the barriers and other predictors in the model.

The predictors of vaccine uptake, after adjusting for other variables, include having some form health insurance (p-value = 0.009), presence of underlying health conditions (p-value = 0.015), and type of healthcare facility visited (UGMC, p-value = 0.006). Participants with health insurance were more likely to take the COVID-19 vaccine as compared with participants without health insurance. This finding corresponded with evidence from a survey by Abbas et al. [1] on the influenza vaccine which indicated that adults without health insurance were less likely not to get vaccinated compared to those with health insurance.

Also, those who accessed healthcare at UGMC were more likely to take the COVID-19 vaccine compared to those who sought healthcare at CCTH. This could be due to attributed to the fact that UGMC was among the first facilities dedicated to individuals infected with COVID-19. Conversely, individuals with underlying conditions were less likely to get vaccinated, compared with those without such conditions.

**Fig. 1:**
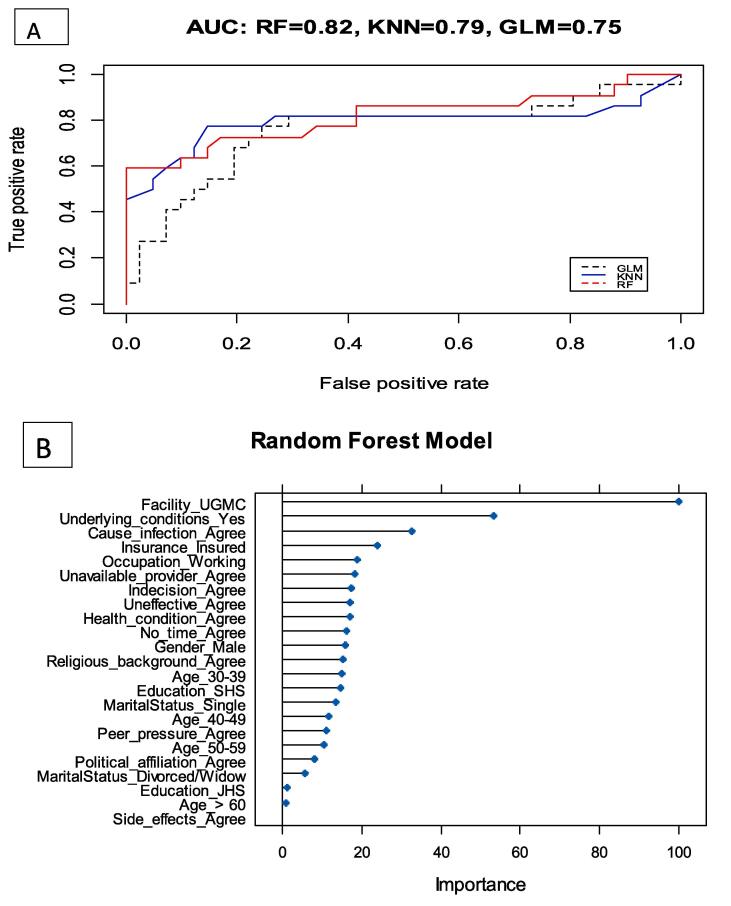
Receiver operative characteristic curve (A) and Importance of Predictive power (B)

Since the logistic regression model cannot identify non-linear relationships and it is non-iterative as well as its dependence on assumptions, other machine learning algorithms were explored. The random forest model reported a higher area under curve (AUC) (0.82) than the KNN model (0.79). The important variables with most predicative power include facility, underlying conditions and perception of COVID-19 vaccine causing infections (Fig. 1).

There are, however, some limitations with this study. The study sites i.e., UGMC and CCTH are quaternary and tertiary facilities respectively; hence, the clients visiting these facilities may differ based on the level of care required and may therefore introduce site-specific biases. It remains to be determined whether this trend will be observed in primary and secondary healthcare facilities. Also, by using a relatively small sample size of 281, there is a limitation on the generalizability of this study as this may not be reflective of the diverse nature of the entire population of Ghana.

## 4 Conclusion

In this study, the results showed that machine learning algorithms can be of great use in determining the uptake of COVID-19 vaccine and possibly other vaccines. Several predictors of vaccine uptake, including barriers and demographics were identified. It was also observed that the type of facility that a client regularly visits and the presence of an underlying health condition played a significant role in determining their likelihood of receiving the COVID-19 vaccine.

**Ethical clearance/approval**.

Ethical clearance was obtained from the University of Health and Allied Sciences Research and Ethics Committee (UHAS- REC A.7 [22] 21 – 20, UHAS-REC A.7 [16] 21–22), the UGMC Institutional Review Board (UGMC-IRB/MRSC/0003/2022) and CCTH Ethics Review Committee (CCTHERC/EC/2022/093).

**Funding**

No external funding was received for this work

## CRediT authorship contribution statement

**Cornelius C. Dodoo:** Conceptualization, Formal analysis, Methodology, Writing – original draft. **Ebo Hanson-Yamoah:** Conceptualization, Methodology, Writing – original draft. **David Adedia:** Formal analysis, Writing – original draft. **Irene Erzuah:** Conceptualization, Methodology, Writing – original draft. **Peter Yamoah:** Writing – original draft. **Fareeda Brobbey:** Methodology, Writing – review & editing. **Constance Cobbold:** Methodology, Writing – review & editing. **Josephine Mensah:** Methodology, Writing – review & editing.

## Declaration of competing interest

The authors declare that they have no known competing financial interests or personal relationships that could have appeared to influence the work reported in this paper.

## Data Availability

Data will be made available on request.
